# Evaluation of asymmetric dimethylarginine as an inflammatory and prognostic marker in dogs with acute pancreatitis

**DOI:** 10.1111/jvim.15785

**Published:** 2020-05-07

**Authors:** Eleonora Gori, Alessio Pierini, Ilaria Lippi, Valentina Meucci, Francesca Perondi, Veronica Marchetti

**Affiliations:** ^1^ Veterinary Teaching Hospital “Mario Modenato,” Department of Veterinary Sciences University of Pisa Pisa Italy

**Keywords:** canine, inflammation, pancreas, severity

## Abstract

**Background:**

Asymmetric dimethylarginine (ADMA) has been proposed as a severity marker in humans with acute pancreatitis (AP).

**Objectives:**

To evaluate ADMA in dogs with AP compared with healthy dogs and its association with severity of disease, mortality, and indicators of the systemic inflammatory response syndrome (SIRS), including serum C‐reactive protein (CRP) concentration, WBC count, and band neutrophils.

**Animals:**

Fifty‐four dogs with AP and a control group (CG) of 28 healthy dogs.

**Methods:**

Cohort study including dogs with AP diagnosed using clinical and laboratory variables, abnormal canine pancreatic lipase (cPL) concentration, and compatible abdominal ultrasound examination findings performed within 48 hours of admission. Canine AP severity (CAPS) was calculated. Serum concentration of ADMA was measured using high performance liquid chromatography. Blood donor‐, staff‐, and student‐owned dogs were enrolled in the CG.

**Results:**

Dogs with AP had higher median admission serum ADMA concentrations compared with the CG (62 versus 48.5 μg/dL; *P* = .003). Dogs with CAPS ≥11 had higher serum ADMA concentrations than did dogs with CAPS <11 (92 versus 54.6 μg/dL *P* = .009). Univariable analysis for mortality, CAPS score, band neutrophils, CRP, and ADMA were included in multivariable logistic regression, in which only ADMA was associated with mortality (*P* = .02). Survivors had a significant decrease in ADMA at first reevaluation compared to admission (*P* = .02).

**Conclusions and Clinical Importance:**

Because serum ADMA concentrations were higher in AP dogs compared with the CG, it may have value as a biomarker in the diagnosis of AP in dogs. In addition, because ADMA was associated with mortality, it may have prognostic value.

**Abbreviations**
ADMA: asymmetric dimethylarginine
AP: acute pancreatitis
CAPS: canine acute pancreatitis severity
CG: control group
cPL: canine pancreatic lipase
CRP: C‐reactive protein
NO: nitric oxide
NOS: nitric oxide species
SIRS: systemic inflammatory response syndrome

## 1 INTRODUCTION

Acute pancreatitis (AP) is the most common disease of the exocrine pancreas in dogs.^1^ It is characterized by a variety of clinical presenting signs, including hyporexia, weakness, vomiting, diarrhea, and abdominal pain. Not only the clinical presentation of AP but also the prognosis depends on the severity of inflammation in the pancreatic parenchyma. Parenchymal inflammation is followed by the release of inflammatory mediators, such as reactive oxygen species, reactive nitrogen species (NOS), and cytokines, which may lead to systemic inflammatory response syndrome (SIRS).^2^ Specifically, during AP, after the initial activation of pancreatic enzymes, local inflammation causes neutrophil migration, and subsequent production of reactive oxygen species (ROS) and NOS, which contribute to ongoing inflammation.^2^ Nitric oxide (NO) is an important molecule that can have regulatory functions in both the circulatory and immune systems. Asymmetric dimethylarginine (ADMA) has been identified as a potent endogenous inhibitor of NO synthesis.^3^ Therefore, an increase in serum ADMA concentration may be an indirect and useful indicator of NOS balance and has been shown to be altered in sepsis.^4^, ^5^, ^6^, ^7^

To the best of our knowledge, no studies in veterinary medicine have evaluated the relationship between ADMA and AP. However, ADMA has been studied in SIRS and sepsis in humans^4^, ^5^ and in cardiovascular diseases in both humans and dogs.^8^, ^9^, ^10^

Our hypothesis was that ADMA would be a marker of disease severity and inflammation in dogs with AP. Our aims were to evaluate serum ADMA concentrations in dogs with AP compared with a control group of healthy dogs (CG) and assess possible relationships between ADMA and severity of disease, acute inflammatory markers (C‐reactive protein, WBC, and band neutrophils) and survival in dogs with AP.

## 2 MATERIALS AND METHODS

Serum ADMA concentrations were evaluated using surplus serum of hospitalized dogs with AP included in a larger prospective study conducted at our veterinary teaching hospital between March 2017 and April 2019, for which ethical approval had been obtained (Approval No. 16749/2017). Surplus serum samples from each dog were frozen at −80°C at the time of sampling (admission to the hospital) pending analysis, which was performed ≤25 months after collection.^11^ Serum concentrations of ADMA are reported to be stable for several years when stored at −70 to −80°C.^11^

A diagnosis of AP was made if all of the following criteria were met: compatible clinical signs (≥2 of the following: abdominal pain, diarrhea, vomiting, hyporexia), changes consistent with AP on abdominal ultrasound examination (Xario XG, Toshiba, Tokyo, Japan) for AP within 48 hours of hospital admission and abnormal SNAP test result for canine pancreatic lipase (cPL) (Idexx Laboratories, Milan, Italy). Abdominal ultrasound examination was performed by an experienced radiologist and was considered consistent with AP if the pancreas was hypoechoic and enlarged, with irregular shape and margins, and surrounded by hyperechoic mesentery or abdominal effusion or both.^12^

In our facility, serum samples from blood donors, and staff, or student‐owned healthy dogs are routinely stored at −80°C for scientific purposes and each owner signs informed consent for such usage. Dogs were considered healthy based on history, physical examination, and blood test results (CBC, serum biochemistry). Twenty‐eight healthy dog serum samples were available for the CG for ADMA analysis.

Each AP dog had a CBC, serum biochemical profile, coagulation profile, urinalysis, and venous blood gas analysis performed as part of routine care for AP. Data from CBC as well as serum creatinine and ionized calcium concentrations at presentation were recorded.

Canine acute pancreatitis severity (CAPS), a recently validated clinical scoring system for short‐term mortality in dogs with AP,^13^ was calculated for each dog at presentation (T0). The previously described cutoff of 11 was used to divide the dogs into 2 groups, because it has been shown to be the most sensitive (89%) and specific (90%) cutoff for survival.^13^

The CAPS score was calculated as follows: CAPS score = 8 × (1 if SIRS, 0 otherwise) + 3 × (1 if coagulation disorders, 0 otherwise) + 4 × (1 if increased serum creatinine concentration, 0 otherwise) + 3 × (1 if ionized hypocalcemia, 0 otherwise).^13^ The CBC was performed using a laser cell counter (Procyte DX, Idexx Laboratories, Westbrook, Maine), and a blood smear stained with May‐Grunwald Giemsa (Aerospray Wescor, Delcon, Milan, Italy) was examined by an experienced clinical pathologist. One hundred WBC were counted and both percentage and absolute band neutrophil count were determined. Serum creatinine and CRP concentrations were measured using serum samples and an automatized biochemistry analyzer (Liasys, Assel SRL, Rome, Italy). Serum ionized calcium concentration was determined using a blood gas analyzer (ABL 700 series, Radiometer Medical, Copenhagen, Denmark). The coagulation profile was performed using an automated coagulation analyzer (Destiny Max, Tcoag Ireland Inc., Wicklow, Ireland). The presence of SIRS was assessed using proposed criteria.^14^ A dog was assigned to the SIRS group if at least 2 of the following 4 criteria were present: hyperthermia or hypothermia (>39.7 or <37.8°C), tachycardia (>160 beats/min), tachypnea (> 40 breaths/min), WBC < 4000/μL or >12 000/μL or band neutrophils >10%.^13^, ^14^

Mortality was evaluated at hospital discharge and dogs were divided into survivors (discharged from the hospital) and nonsurvivors (died or euthanized for the worsened clinical condition despite treatment).

Approximately 2 weeks (10‐20 days) after discharge, survivors were clinically reevaluated. A CBC and serum biochemical profile were performed, and surplus serum was stored at −80°C until analysis.

Serum ADMA concentrations were measured both at T0 (hospital admission) and at the first reevaluation (T1). Serum concentrations of ADMA were determined using high performance liquid chromatography with fluorescence detection, as previously described.^11^ Briefly, blood was centrifuged immediately after collection, and serum was frozen at −80°C and stored until analysis. For serum ADMA concentration, the intra‐ and interassay coefficients of variation were <9 and <11%, respectively. The lower limit of quantification, at a signal‐to‐noise ratio of 10, was 5 μg/dL for ADMA using a 0.2 mL sample volume.

### 2.1 Statistical analysis

All continuous variables were tested using the Kolmogorov‐Smirnov normality test. Normally distributed variables were expressed using the mean ± SD, and nonnormally distributed variables were expressed using the median and range.

The T0 serum ADMA concentration was compared between AP dogs and CG using a Mann‐Whitney *U* test.

A Mann‐Whitney *U* test was performed to determine if T0 ADMA was different between dogs with and without SIRS and between CAPS score groups (< or ≥11). Spearman's or Pearson's correlation test was performed between ADMA and serum C‐reactive protein (CRP), WBC, and band neutrophil count.

A univariable analysis was performed to identify variables associated with mortality using t‐tests or the Mann‐Whitney *U* test based on data distribution (age, weight, WBC, band neutrophil count, CRP, and T0 ADMA) or chi‐square test (CAPS score groups and SIRS). All variables with a *P*‐value <.2 were subjected to multivariable analysis. Afterward, multivariable backward stepwise binary logistic regression was performed to assess the association of variables with mortality, using the variables previously identified in the univariable model as significantly associated with mortality. If a variable remained significantly associated with mortality (*P* < .05), it was considered to be associated with the death of the dog.

Finally, T1 serum ADMA concentrations in survivors were compared with T0 ADMA (paired *t* test) to establish if, after remission of AP, survivors had undergone a significant change in serum ADMA concentration.

Data were analyzed using commercial software (IBM SPSS Statistics, version 25, IBM Corporation, New York, New York; GraphPad Prism, version 7.0a, GraphPad Software Inc, San Diego, California).

## 3 RESULTS

### 3.1 Study population

Fifty‐four dogs diagnosed with AP were enrolled with the owners’ informed consent. Mean age was 10.4 ± 3.5 years. The most commonly affected breeds were Poodle (n = 4), German Shepherd (n = 2), Lagotto Romagnolo (n = 2), Labrador Retriever (n = 2), Beagle (n = 2), Jack Russell terrier (n = 2), Springer Spaniel (n = 2), Shih Tzu (n = 2), and Spitz (n = 2). Seventeen dogs (33%) were mixed breed and the remaining 17 dogs belonged to other breeds (one each of Bloodhound, Border Collie, Bouvier des Flandres, Boxer, Cavalier King Charles Spaniel, Cocker Spaniel, Dachshund, English Setter, Epagneul Breton, Flat Coated Retriever, French and English Bulldog, Golden Retriever, Great Dane, Italian Pointer, Pug, Yorkshire Terrier). Twenty‐five of 54 dogs (46.3%) were females, of which 8 were spayed, whereas the other 29 dogs were males (53.7%), of which 12 were castrated. The median weight was 16.5 kg, ranging from 3.27 to 40.7 kg. In our cohort of dogs, the CAPS score ranged from 0 to 15 with a median of 8. One dog had a CAPS score of 3, 31 dogs had CAPS scores of 8, whereas 5 and 8 dogs had CAPS scores of 11 and 12, respectively. Seventeen dogs (31.5%) were in the nonsurvivor group. The CG included 28 mixed breed dogs 4.5 ± 4 years of age. Mean weight of the CG was 26 ± 19 kg.

### 3.2 Serum ADMA concentrations between AP dogs and CG and association between ADMA and CAPS score and SIRS

**FIGURE 1:**
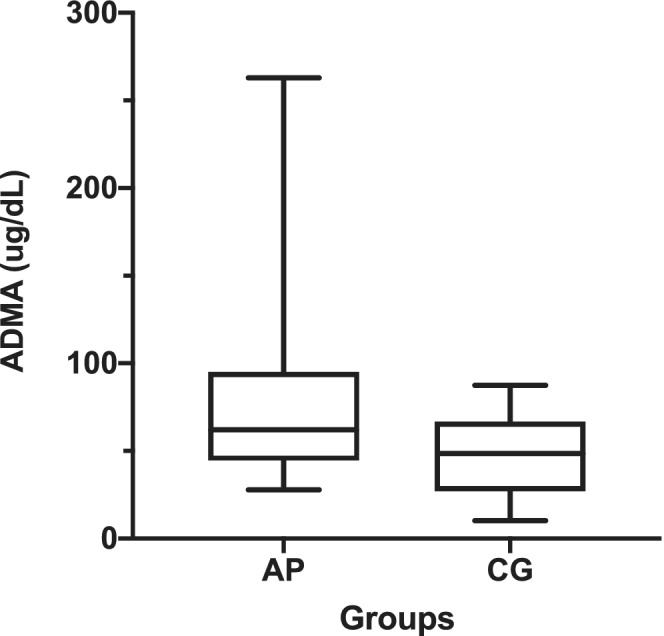
Box and whisker plot comparing median serum ADMA concentrations between dogs with acute pancreatitis (AP) and healthy dogs (CG). The line represents the median, box depicts the 25th and 75th percentiles, and the whiskers express the range of data. There is considerable overlap in the data between the 2 groups although the median concentration of ADMA of the AP group is significantly higher than CG (Mann‐Whitney *U* test, *P* = .003)

**FIGURE 2:**
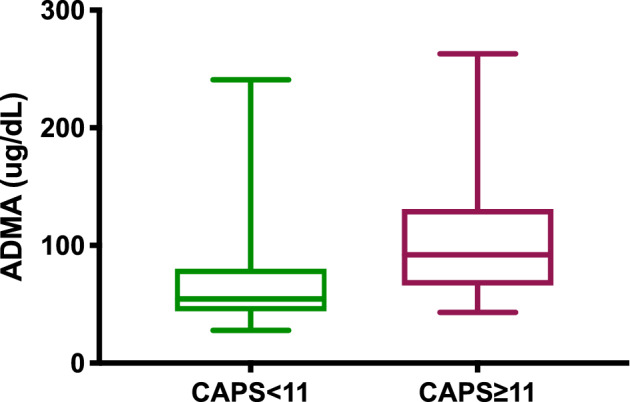
Box and whisker plot comparing serum ADMA concentrations between dogs with acute pancreatitis divided into groups using the previously validated canine acute pancreatitis severity (CAPS) cutoff^12^ (CAPS ≥11 and <11). Dogs with CAPS ≥11 showed a significantly higher median ADMA concentration than dogs with CAPS<11 (Mann‐Whitney *U* test, *P* = .009)

The AP dogs had significantly higher median serum ADMA concentration compared with the CG (62 μg/dL; interquartile range [IQR], 50.51 versus 48.5 μg/dL; IQR, 39.8; *P* = .003; Figure 1). Those AP dogs with CAPS ≥11 had higher serum concentrations of ADMA than did dogs with CAPS <11 (92 μg/dL; IQR, 64.7 versus 54.6 μg/dL; IQR, 36.2; *P* = .009; Figure 2). The SIRS was present in 28 dogs (51.8%). No difference in median serum ADMA concentration was found between dogs with and without SIRS (56.3 versus 68.4 μg/dL; *P* = .24). In addition, no significant correlation was found between serum ADMA concentration and CRP, WBC, or band neutrophil count (*P* = .35, *P* = .83, and *P* = .38, respectively).

### 3.3 Mortality analysis

The univariable analysis results are shown in Table 1. In the binary logistic regression analysis including band neutrophils, CRP, serum ADMA concentrations, and CAPS score, only serum ADMA concentration was significantly associated with survival (*P* = .02; Table 2).

**TABLE 1:** Univariable analysis of selected parameters between survivors (n = 37 dogs) and nonsurvivors (n = 17)

Variable	Survivors (n = 37)	Nonsurvivors (n = 17)	*P*‐value
Age (years)	10.3 ± 3.74	10.63 ± 3.05	.84
Weight (kg)	14.4 (3.3‐37)	23.5 (3.27‐40)	.27
CAPS score	8 (0‐15)	8 (0‐12)	**.08**
CAPS score ≥ 11	6/37	8/17	**.02**
WBC (K/μL)	11.8 (5‐49.9)	14.7 (0.7‐87.9)	.61
Band neutrophils (K/μL)	0 (0‐7.15)	0.31 (0‐11.3)	**.11**
CRP (mg/dL)	0.8 (0–12)	1.4 (0.3‐5.6)	**.04**
Presence of SIRS	18/37	10/17	.49
T0 ADMA (μg/dL)	55.1 (27.8‐170)	94.6 (31.6‐262.9)	**.02**

*Note*: Normally distributed variables were expressed using mean ± SD, while the nonnormally distributed variables were expressed using median and range. The variables in bold are those that were entered into the multivariable logistic regression procedure after elimination of variables with a univariable mortality association of *P* > .20.

Abbreviations: ADMA, asymmetric dimethylarginine; CAPS, canine acute pancreatitis severity; CRP, c‐reactive protein; SIRS, systemic inflammatory response syndrome; WBC, white blood cells.

**TABLE 2:** Results of the multivariable backward stepwise binary logistic regression model for mortality

		B	SE	Wald	dF	Sig.	OR	Lower 95% OR	Upper 95% OR
Phase 1	CAPS	.102	.100	1.030	1	.31	1.107	.910	1.347
	BN	.297	.204	2.122	1	.14	1.346	.920	2.008
	CRP	.083	.153	.295	1	.59	1.087	.805	1.467
	ADMA	.012	.008	2.519	1	.11	1.013	.997	1.028
Phase 2	CAPS	.115	.097	1.393	1	.24	1.122	.927	1.358
	BN	.294	.204	2.072	1	.15	1.342	.855	2.004
	ADMA	.013	.008	2.625	1	.10	1.013	.997	1.025
Phase 3	BN	.267	.199	1.807	1	.18	1.306	.885	1.929
	ADMA	.014	.008	3.475	1	.002	1.014	.999	1.030
Phase 4	ADMA	.016	.007	5.501	1	.02	1.016	1.003	1.030

Abbreviations: ADMA, asymmetric dimethylarginine; B, B statistic; BN, band neutrophils; CAPS, canine acute pancreatitis severity; CRP, c‐reactive protein; dF, degrees of freedom; OR, odds ratio; Sig., significance; Wald, Wald statistic.

### 3.4 Follow‐up analysis

Sixteen of 37 serum samples (43.2%) were available for determination of T1 serum ADMA concentration. All of the survivors had resolution of the clinical signs of AP and a significant decrease in T1 compared with T0 serum ADMA concentrations (median, 44.6 versus 73.3 μg/dL; *P* = .02).

## 4 DISCUSSION

In AP in dogs, serum ADMA concentration is associated with the severity of the disease and mortality. The serum ADMA concentrations in dogs with AP and healthy control dogs, respectively, had broad overlap but no healthy animal had serum ADMA concentration that exceeded 100 μg/dL.

Many studies in humans have taken serum ADMA concentration into account, which plays an important regulatory role in the synthesis of NO from L‐arginine.^11^ Unlike symmetric dimethylarginine (SDMA), which is almost exclusively excreted by the kidneys, ADMA is mainly metabolized by the enzyme dimethylarginine dimethylaminohydrolase in the cytosol of liver and kidney tissue.^4^ In humans and experimental animals, ADMA concentration is a well‐known marker of endothelial dysfunction, and increased concentrations are associated with hypercholesterolemia, hypertension, diabetes and other conditions, including SIRS and sepsis.^4^, ^5^, ^6^, ^7^, ^11^ Serum ADMA concentration also is a recognized marker of cardiac diseases in humans, and it is significantly increased in humans with valvular disease or idiopathic cardiomyopathy.^11^

However, compared to human medicine, in small animals the clinical and prognostic relevance of serum ADMA concentration still is unclear. In 2 studies, serum ADMA concentration was significantly increased in Beagles with experimentally induced congestive heart failure.^8^ A more recent study, performed in Cavalier King Charles Spaniels with various degrees of severity of myxomatous mitral valve disease, failed to find an association between serum ADMA concentration and the severity of the cardiac disease.^10^

In our study, serum ADMA concentration was significantly higher in nonsurvivors and in dogs with more severe disease (CAPS >11), but it was not associated with the presence of SIRS or correlated with inflammatory markers commonly used in clinical practice (CRP and WBC). Furthermore, serum ADMA concentrations were significantly lower in the survivor group, after resolution of AP.

Serum ADMA concentration already has been reported as a marker of endothelial dysfunction in dogs, and nonsurvivors may have more severe oxidative stress, which may lead to an increase in serum ADMA concentration. However, we failed to find any association between serum ADMA concentration and SIRS, CRP, or WBC. This perhaps was because serum ADMA concentration appeared to be more related to oxidative stress status than to SIRS itself.^2^, ^13^ As in human medicine, where serum ADMA concentration has been linked to inflammatory status and prognosis,^4^, ^5^, ^6^, ^7^, ^15^ ADMA may be involved in the inflammatory and oxidative stress balance in dogs with AP. However, it is possible that serum ADMA concentration will be increased in some dogs that have a predisposition to a more severe compensatory anti‐inflammatory response syndrome, which can exist separately from SIRS, as a distinct set of cytokines is involved.^16^

Serum ADMA concentration was studied primarily in septic human patients, in which it recently was identified as a prognostic marker.^5^ One study showed that the occurrence of high plasma ADMA concentrations identified individuals at risk of death and who might need intensive care.^5^

Our study had several limitations. In the follow‐up analysis, serum ADMA concentrations were measured in only 16 dogs of the survivor group, and it would have been valuable to have a larger study population. Secondly, other measurements of oxidative stress, such as NOS, reactive oxygen metabolites, and biological antioxidant potential could have provided more information on the oxidative stress status in our dogs and, thus, a better understanding of the role of serum ADMA concentration in dogs with AP. Thirdly, we chose to evaluate serum ADMA concentration in relation to SIRS and other widely used inflammatory markers as WBC, CRP and band neutrophils, however, it could be interesting to study ADMA in relation to other markers, such as cytokines. Breed, age, and sex have not been explored in relation to serum ADMA concentration. Finally, naturally occurring AP in dogs is very often accompanied by or caused by other conditions. However, such comorbidities could not necessarily be ruled out, and they were not taken into account in evaluating mortality.

In conclusion, our study suggests an association between high serum ADMA concentration and death in dogs with AP. Finally, as reported in humans, in our study serum ADMA concentration was significantly decreased over time in dogs that survived.

## CONFLICT OF INTEREST DECLARATION

Authors declare no conflict of interest.

## OFF‐LABEL ANTIMICROBIAL DECLARATION

Authors declare no off‐label use of antimicrobials.

## INSTITUTIONAL ANIMAL CARE AND USE COMMITTEE (IACUC) OR OTHER APPROVAL DECLARATION

The study was approved by the Scientific Ethical Committee for Animal Testing of the University of Pisa Approval No. 16749/2017.

## HUMAN ETHICS APPROVAL DECLARATION

Authors declare human ethics approval was not needed for this study.
